# A novel method to monitor COVID-19 fatality rate in real-time, a key metric to guide public health policy

**DOI:** 10.1038/s41598-022-23138-4

**Published:** 2022-10-31

**Authors:** Yuanke Qu, Chun Yin Lee, K. F. Lam

**Affiliations:** 1 Department of Statistics and Actuarial Science, The University of Hong Kong, Hong Kong, People’s Republic of China; 2 Department of Applied Mathematics, The Hong Kong Polytechnic University, Hong Kong, People’s Republic of China; 3 Centre for Quantitative Medicine, Duke-NUS Medical School, Singapore, Singapore; 4 Guangdong Ocean University, Zhanjiang, People’s Republic of China

**Keywords:** Epidemiology, Infectious diseases

## Abstract

An accurate estimator of the real-time fatality rate is warranted to monitor the progress of ongoing epidemics, hence facilitating the policy-making process. However, most of the existing estimators fail to capture the time-varying nature of the fatality rate and are often biased in practice. A simple real-time fatality rate estimator with adjustment for reporting delays is proposed in this paper using the fused lasso technique. This approach is easy to use and can be broadly applied to public health practice as only basic epidemiological data are required. A large-scale simulation study suggests that the proposed estimator is a reliable benchmark for formulating public health policies during an epidemic with high accuracy and sensitivity in capturing the changes in the fatality rate over time, while the other two commonly-used case fatality rate estimators may convey delayed or even misleading signals of the true situation. The application to the COVID-19 data in Germany between January 2020 and January 2022 demonstrates the importance of the social restrictions in the early phase of the pandemic when vaccines were not available, and the beneficial effects of vaccination in suppressing the fatality rate to a low level since August 2021 irrespective of the rebound in infections driven by the more infectious Delta and Omicron variants during the fourth wave.

## Introduction

Emerging infectious diseases appear more frequently worldwide. For example, the severe acute respiratory syndrome (SARS) in 2003, the Middle East respiratory syndrome (MERS) in 2013, the Ebola virus disease (EVD) in 2014 and the most recent threat, novel coronavirus disease (COVID-19), all pose tremendous challenges to public health globally. In particular, the COVID-19 pandemic continues to spread around the world and has caused sustained outbreaks across 221 countries and territories, resulting in more than 5.4 million deaths out of nearly 289 million confirmed cases as of January 2, 2022^1^. Due to the lack of knowledge and inadequate preparedness to combat new viruses, outbreaks of these novel epidemics often hit affected areas hard, not only as a health crisis in the short term but also as a devastating social and economic disruption in the long term. In particular, the quantification of disease severity is of great concern for public health officials to assess the risk of epidemics and make informed decisions.

The case fatality rate (CFR), calculated as the ratio of cumulative deaths to confirmed cases, is presumably the most commonly used epidemiological quantity for measuring disease severity. Although the crude CFR has a simple interpretation and only requires minimal data inputs, it has two important limitations in practice. First, it assumes that the underlying disease severity is constant over time. Second, it takes no account of the time delay from disease onset to death. In the context of the current COVID-19 pandemic, however, (i) the epidemic has lasted for a long time, approximately two years; (ii) virulent mutant variants of COVID-19 have emerged since 2021^2^; (iii) there is a significant reporting delay in time from disease onset to death^3^; and (iv) a non-negligible proportion of the population are getting vaccinated worldwide^4^. Therefore, the disease severity no longer depends solely on disease virulence, but varies according to a basket of time-varying confounding factors^5^. For instance, a worsening fatality rate always informs public health professionals to adopt timely response strategies, such as social distancing or travel bans, before the hospitals are slammed by infected patients. On the other hand, an improvement in the fatality rate is expected to appear shortly after effective measures are taken, such as the introduction of a specific treatment or a boosted vaccination rate in the population. Hence, the CFR which assumes the underlying disease severity is constant over time with no adjustment for the reporting delay can cause severe bias in practice.

Several methods have been proposed in the literature to adjust for the reporting delays in the CFR estimator. Specifically, the numerator of CFR comprises only the reported deaths but it ignores the fact that some active cases that are part of the denominator will eventually die but may not be observed at the time of analysis, known statistically as censoring. The shrinkage in the numerator results in a downward bias in the estimation of the fatality rate, especially in the early phase of the outbreak. To address the censoring problem in the CFR estimator, a modified Kaplan-Meier approach^6^ and a parametric mixture model^7^ have been proposed using survival analysis techniques to obtain the fatality rate of the 2003 SARS epidemic, respectively. However, these methods typically require high-quality survival data, which makes them applicable only to a small number of hospitalized cases for which the individual-level data are available. In nationwide epidemiological studies, tracking the status of each infected individual induces unrealistic administrative costs, if not impossible. Alternative methods have been proposed to correct the downward bias by adjusting the denominator of the crude CFR with the consideration of the distribution of time from disease onset to death^8–11^. In these adjustments, the numerator is unchanged but the denominator is restricted to the predicted number of cases adjusted for the delay from disease onset to death, the distribution of which can be informed by some prior knowledge or by analyzing some existing hospitalized cases.

All the aforementioned estimators assume that the underlying fatality rate is constant throughout the epidemic with an aim to obtain an overall disease severity at the end of the epidemic, but they pay less attention to the changes in fatality rate over the course of an epidemic. To better cope with the time-varying nature of the underlying fatality rate, different real-time estimators have been proposed in the literature through modeling the transition from disease onset to the final outcomes (death or recovery), under the competing risks framework^6,7,12,13^. These methods work reasonably well for certain diseases when the time to death is comparable to the time to recovery, as in the 2003 SARS epidemic, but they tend to be biased if the time to death and time to recovery have different distributions^14,15^. Hence, it limits the use of these methods in some epidemics, like the ongoing COVID-19 epidemic, because the time from disease onset to death is typically shorter than that to recovery^16^.

In this article, we propose a real-time fatality rate estimator adjusted for reporting delay (rtaCFR) which is able to capture the changes in fatality rate due to a variety of distinguishable factors, such as the implementation of effective health policies or treatments, as well as some indistinguishable factors like a new mutant variant and weather. It provides policymakers with rich information on decision making process. The proposed method is simple, widely applicable to public health practice, and requires only basic epidemiological data, i.e., the cumulative numbers of cases and deaths over time. Simulation results demonstrate the strength of the proposed method in terms of high accuracy and sensitivity to capture the changes in disease severity over time. The proposed estimator is shown to be empirically unbiased in all scenarios, whereas the two commonly used fatality rate estimators perform well only when the underlying fatality rate is constant over time; otherwise, they may convey delayed or even misleading information about the trends in disease severity. We illustrate the usefulness of the proposed method through an application to the COVID-19 data in Germany from January 2020 to January 2022.

## Methods

### Setting

We assume that minimal epidemiological data are collected on a regular basis during an ongoing emerging epidemic. For a given observation time point *t*, the observed numbers of deaths and confirmed cases reported on day *j* are denoted by *d*(*j*) and *c*(*j*), respectively, for $j=1,\dots ,t$. Suppose that the final outcome for each diagnosed case is either death or recovery. We denote *F* as the cumulative distribution function of the time from disease onset to death with $F(0)=0, F(S)=1$, $0\le F(s)\le 1$ for $0\le s\le S$, and *S* pertains to the maximum day to incur death given the onset of illness. Here, *F* can be informed by prior knowledge, such as data obtained from past outbreaks, or through analysis of some hospitalization cases for which the individual-level data are available.

### Existing CFR estimators

Subject to the minimal data available in the contexts of emerging diseases, there are two commonly used fatality rate estimators. The first one is the traditional CFR calculated as the ratio of the cumulative number of deaths to the cumulative number of confirmed cases, given by

1 $$\begin{aligned} \text {CFR}(t) = \frac{\sum _{j=1}^{t} d(j)}{\sum _{j=1}^{t}c(j)}. \end{aligned}$$

The second one is the time-delay adjusted case fatality rate (aCFR) proposed by Nishiura et al.^8^, which is formulated as

2 $$\begin{aligned} \text {aCFR}(t) = \frac{\sum _{j=1}^{t} d(j)}{\sum _{j=1}^{t}c(j)F(\min (t-j,S))}, \end{aligned}$$

where $\min (a,b)=a$ if $a<b$. It is remarked that the first estimator does not adjust for the delay from disease onset to death, but the second one takes this into account by multiplying an adjustment factor $F(\min (t-j,S))$ to the components in the denominator. However, both estimators assume that the underlying fatality rate is constant over a given period [0, *t*) and aim to estimate the overall fatality rate up to time *t*, thus failing to reflect the changes in disease severity over time.

### The proposed real-time fatality rate estimator

We model *p*(*j*) as the proportion of confirmed cases reported on day *j* who will eventually die from the disease. In practice, *p*(*j*) is potentially influenced by many factors including but not limited to virus virulence, treatment effect and quality of healthcare. The primary goal of our study is to estimate *p*(*j*), the real-time fatality rate adjusted for reporting delay, for $j=1,\dots ,t$, and we denote the estimator of *p*(*j*)’s as rtaCFR(*t*). Under this modeling framework, the expected number of observed deaths on day *j* is

3 $$\begin{aligned} {\mathbb {E}} [d(j)]=\sum _{s=0}^{j-1}p(j-s)c(j-s)f(s+1), \qquad j=1,\dots ,t, \end{aligned}$$

where $f(s)=F(s)-F(s-1)$ for $1\le s\le S$. Specifically, among the confirmed cases reported on day $j-s$, it is expected that $p(j-s)c(j-s)$ of them will ultimately die from the disease according to the Bernoulli process with probability $p(j-s)$, and $f(s+1)$ governs the time delay for the deaths to be observable at time *j* for $s=0,\dots ,j-1$. Note that ${\mathbb {E}} [d(j)]$ is purely a function of $p(1),\dots ,p(j)$ given the distribution function *F* and the observed series of confirmed cases $c(1),\dots ,c(j)$, for $j=1,\dots ,t$. Hence, equation (3) is a standard linear regression model which can be rewritten into matrix form as

4 $$\begin{aligned} \left[ \begin{matrix} {\mathbb {E}}[d(1)] \\ {\mathbb {E}}[d(2)]\\ \vdots \\ \vdots \\ \vdots \\ {\mathbb {E}}[d(t)] \end{matrix}\right] =\left[ \begin{matrix} f(1) &{} 0 &{} \cdots &{} \cdots &{} \cdots \ {} &{} 0\\ \vdots &{} \ddots &{} &{} &{} &{}\vdots \\ f(S) &{} &{} \ddots &{} &{} &{} \vdots \\ 0 &{} f(S) &{} \cdots &{} f(1) &{} \cdots &{} 0\\ \vdots &{} &{} \ddots &{} &{} \ddots &{}\vdots \\ 0&{} \cdots &{} 0 &{} f(S) &{} \cdots &{} f(1) \end{matrix} \right] C \left[ \begin{matrix} p(1) \\ p(2)\\ \vdots \\ \vdots \\ \vdots \\ p(t) \end{matrix} \right] \end{aligned}$$

where $C=\texttt {diag}(c(1),\dots ,c(t))$, and $\texttt {diag}$ denotes the diagonal matrix operator. It is easy to see that when $p(1)=p(2)=\dots =p(t)=p$ in (4), the estimate for *p* at time *t* in (3) is equivalent to the aCFR(*t*) in (2) as they are estimating the same quantity, the constant fatality rate over the period [0, *t*).

Considering the temporal structure in (4), the fused lasso technique with fusion penalty can be applied to obtain rtaCFR(*t*) $=\left( {\widehat{p}}(1), {\widehat{p}}(2), \ldots , {\widehat{p}}(t)\right)$ by solving the following minimization problem^17,18^

5 $$\begin{aligned} \text {rtaCFR}(t)=\underset{p \in [0,1]^{t}}{{\text {argmin}}}\, \frac{1}{2} \sum _{j=1}^{t}\left( d(j)-{\mathbb {E}}[d(j)]\right) ^{2}+\lambda \sum _{i=1}^{t}\left| p(i)-p(i-1)\right| , \end{aligned}$$

where $\lambda$ is a non-negative tuning parameter penalizing the absolute differences in successive coordinates of *p* as the values of fatality rate within a short time interval are closely related to each other. When $\lambda =0$, the penalty term has no effect, and the fused lasso will produce the classical least squares estimates. As $\lambda$ increases, the resulting ${\widehat{p}}$’s of adjacent time points are shrunken towards each other, and the estimates become homogeneous as $\lambda$ approaches infinity. In practice, $\lambda$ is chosen with the smallest residual sum of squares subject to $0 \le {\widehat{p}}(j)\le 1$ for all $j=1,\dots ,t$. The computation can be performed via the R package genlasso^19^, which provides solutions for all values of the tuning parameter $\lambda$ with the associated residual sum of squares values.

### Ethics approval

Not applicable (no human subjects used), as we preformed the study from publicly available data.

## Simulation studies

**Figure 1:**
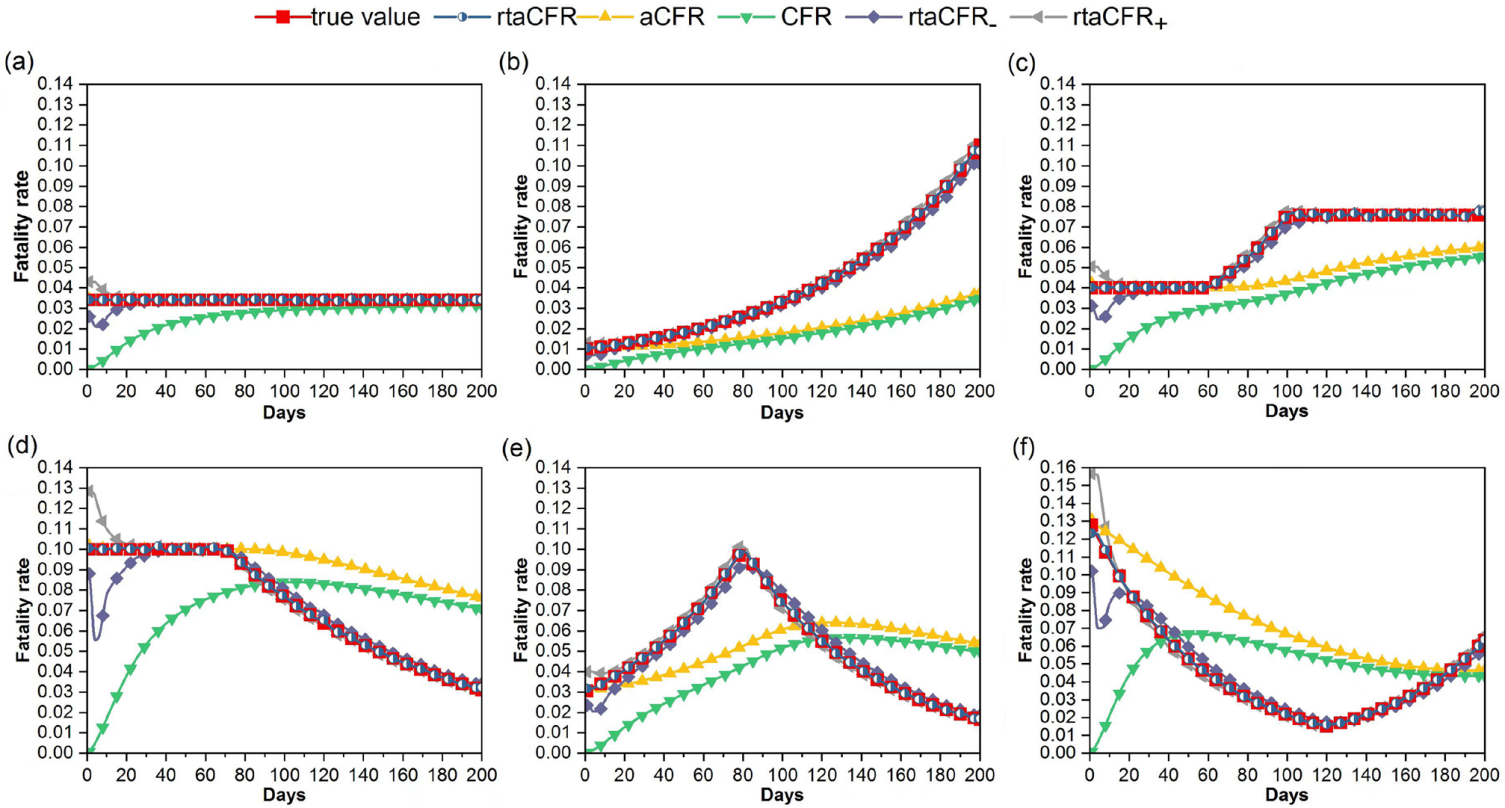
Performance of the proposed real-time fatality rate estimator (rtaCFR) ($\textrm{rtaCFR}_{-}$ and $\textrm{rtaCFR}_{+}$ are for robustness check) compared with the traditional case fatality rate (CFR) and the time-delay adjusted case fatality rate (aCFR) under different hypothetical scenarios of true values based on 1000 replicates. The true values are (**a**) $p(t)=0.034$; (**b**) $p(t)=0.01\cdot e^{0.012t}$; (**c**) $0.04\cdot e^{0.016\cdot \text {I}(t>60) \cdot \text {min}(40,t-60)}$; (**d**) $0.1\cdot e^{-0.009\cdot (t-70)\cdot \text {I}(t>70)}$; (**e**) $0.1\cdot e^{-0.015\cdot |t-80|}$; and (**f**) $0.015\cdot e^{0.018\cdot |t-120|}$ for $t=1,\ldots ,200$.

We compare the performance of the proposed rtaCFR with those based on CFR and aCFR using simulated data under various hypothetical scenarios. We assume that the epidemiological data set contains only the cumulative numbers of confirmed cases and deaths, and that the exact time of infection and death for each case is generally unknown. This mimics the actual situation of most epidemic outbreaks that the individual-level data are not readily accessible or completely missing, especially for the areas with a weak surveillance system. We set a 200-day observation period and set the daily number of confirmed cases to be $c(t)=3000-5\cdot |100-t|$, for $t=1,\ldots ,200$. This mimics an outbreak with an initial surge in the number of confirmed cases followed by a decline in infections when control strategies are implemented. We then consider six scenarios with different patterns of *p*(*t*), namely (a) constant fatality rate; (b) exponentially increasing fatality rate; (c) constant fatality rate at both ends with a linearly increasing rate from day 60 to 100; (d) constant fatality rate followed by an exponentially decline; (e) exponentially increasing followed by exponentially decreasing fatality rate; and (f) exponentially decreasing followed by exponentially increasing fatality rate. The values of the time-varying *p*(*t*) in each scenario are represented by red lines with squares in Fig. 1a–f, respectively.

Given the specified series of *c*(*t*) and *p*(*t*) under each scenario, the numbers of deaths on day *t* namely *d*(*t*) can be simulated. Specifically, for the confirmed cases reported on day *t*, they will eventually die of the disease with a probability of *p*(*t*), and the time from onset to death is determined by *F*. In the simulation setting, *F* is chosen to be the distribution function of a gamma random variable with mean $\mu =15.43$ days and shape parameter $\gamma =2.03$, which pertains to the situation of COVID-19 outbreak estimated by a recent study^20^. It follows that the rtaCFR can be computed based on (5) given *F*, simulated confirmed cases and deaths. To compare the performance of the proposed rtaCFR, with aCFR and CFR, Fig. 1 provides the averaged estimates constructed based on 1000 replicates under the six hypothetical situations. We observe the following patterns in our simulations. First, at the beginning of the observation period, the CFR is subject to a downward bias whereas aCFR and rtaCFR are nearly unbiased. It is due to the fact that CFR does not adjust for time delay from onset to death, unlike the other two estimates. Second, when the true fatality is constant over time (i.e. Fig. 1a, and early period of Fig. 1c,d, both aCFR and rtaCFR are nearly unbiased. This result is not surprising as the rtaCFR actually includes the aCFR as a special case when the underlying fatality rate is constant. Third, when the true fatality is not constant, rtaCFR is still able to pick up the changes in the fatality rate over time in the sense that the red and blue lines align with each other closely, but large disparities are seen for CFR and aCFR that the bias cannot be remedied by prolonging the observation period. Presumably, the insensitivity of the latter two estimators can be attributed to their constant fatality rate assumption.

Importantly, the simulation results suggest that it is inappropriate to use CFR and aCFR estimates as a guideline for implementing certain public health policies during an epidemic as they may show a misleading trend or even a trend contrary to the truth. Scenario (e) corresponds to the case where the fatality rate of the disease increases in the early phase due to lack of preparedness and then drops gradually after a certain time point, which could be a result of introducing an effective treatment or increasing hospital capacity. We can see that both the CFR and aCFR are insensitive to pick up the decreasing trend of fatality rate in the late phase of the epidemic, therefore, fail to detect the positive effect of certain treatments or implemented policies. On the other hand, scenario (f) mimics the situation of the ongoing COVID-19 epidemic in most countries, where the fatality rate initially declines and then increases sharply with a new wave of infections probably due to the easing of public health measures or the emergence of a more virulent mutant variant. We can see that the proposed rtaCFR has an excellent performance in capturing changes in disease severity over time, whereas the other two estimators present a false increasing trend in the early stages and then remain roughly constant throughout the epidemic period.

To evaluate the robustness of the proposed method against the specification of *F*, we also fit the proposed model to the simulated data using 10 days^9,21^ and 18 days^22^ as the mean length from disease onset to death. The corresponding estimates are labelled as $\textrm{rtaCFR}_{-}$ and $\textrm{rtaCFR}_{+}$ respectively, in Fig. 1. We can see that although a small disparity is observed among $\textrm{rtaCFR}, \textrm{rtaCFR}_{-}$ and $\textrm{rtaCFR}_{+}$ in the early stage, the three estimates are almost consistent and unbiased for the time-varying disease severity. It shows that the proposed method is quite robust with a reasonable range of distributions. In addition, we have tried different sequences of daily number of confirmed cases in the simulation setup, such as $c(t)=3000+5t$ and $c(t)\approx 800$, as well as using the number of confirmed cases in Germany for the latest 200 days up to the date of writing on 7 January 2022. The results obtained are quite robust to these changes, hence are not reported here.

### The COVID-19 pandemic in Germany

**Figure 2:**
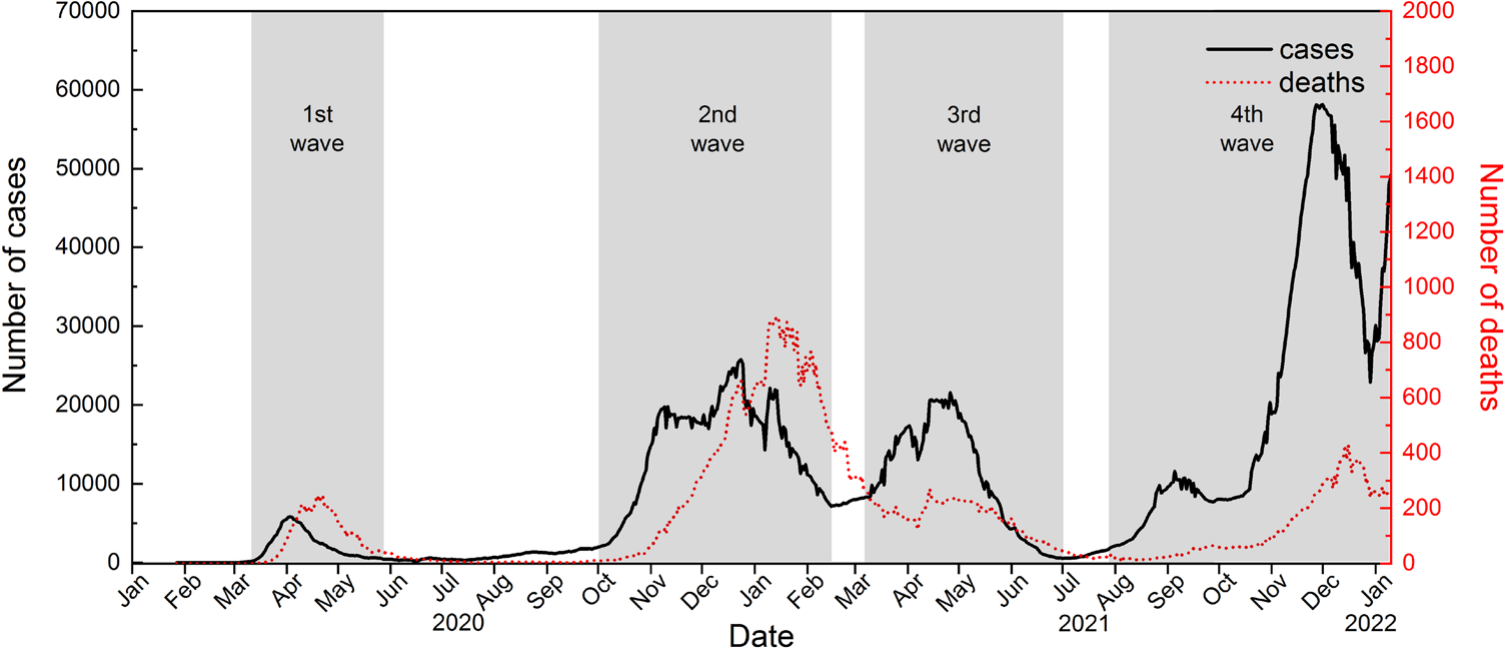
Seven-day moving average of daily numbers of confirmed cases and deaths in Germany from 27 January 2020 to 7 January 2022.

We illustrate the proposed adjusted real-time fatality rate estimator using COVID-19 data from Germany, which is known as a role model for epidemic management as compared to other worst-hit European countries, especially in the early stages of the epidemic^23,24^. Epidemiological data for daily series of cases and deaths from the time of the first incidence in Germany reported on 27 January 2020 to the date of writing, 7 January 2022 were extracted from the public domain^25^. The seven-day moving averages of daily numbers of confirmed cases and deaths are plotted in Fig. 2.

**Figure 3:**
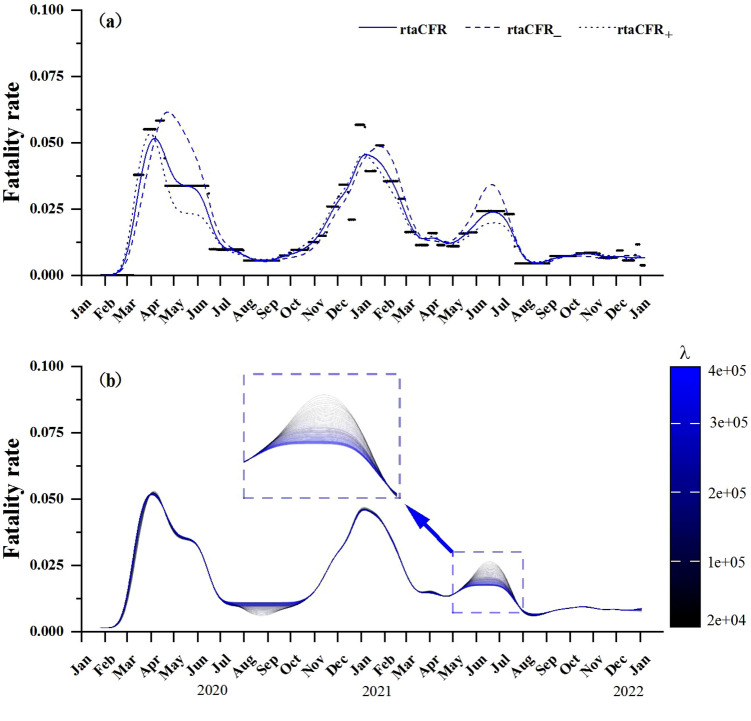
Estimation for the COVID-19 data in Germany (**a**) original rtaCFR (piecewise constant black lines) plus smoothed rtaCFR (blue lines) based on different Gamma distributions for *F*, using the optimal tuning parameter; (**b**) smoothed rtaCFR obtained based on different tuning parameter values for sensitivity analysis.

The first infection in Germany was reported on 27 January 2020 and the number of confirmed cases escalated rapidly since then^26^. In response to the unprecedented pandemic, a crisis management team was set up and a series of precautionary measures, including school closure, social distancing and travel bans, were implemented in Germany since March 2020^27–29^. Owing to the effective containment strategies and a lockdown since 22 March 2020, the number of infections in Germany dropped substantially in the weeks that follow^30^. In view of the significant decline in incidence, the government decided to gradually ease the lockdown restrictions starting from 4 May 2020. However, after a few months of the holiday season without strict public health measures, Germany encountered a second wave of infections. We can see from Fig. 2 that the daily number of infections in October 2020 returned to the peak level as in April of the same year. While other European countries imposed a strict lockdown in early November 2020 to control the disease, Germany adopted a softer lockdown, named as ‘lockdown light’, to deal with the second wave of the disease^31^. The partial lockdown started on 2 November 2020 with restaurants, bars, gyms and entertainment venues closed, but schools and most businesses were allowed to remain open in the hope of striking a balance between public health and the economy. However, the partial lockdown failed to stop the spread of the disease until a hard lockdown was declared in December 2020^32^. Since then, a vaccination program prioritizing the elderly also began^33^. We can see from Fig. 2 that there was a temporal decline in the trend of confirmed cases from January to February 2021, but the infection figure bounced back in March, possibly caused by the emergence of the Alpha variant, a more contagious strain of COVID-19. In August 2021, the Delta variant and the gradual easing of social restrictions led to the fourth wave of infections in Germany, and the new fastest-spreading Omicron variant has further driven up the infections since the end of 2021^34^. On the contrary, the trend of the death toll was fairly simple, with literally three peaks recorded in early April 2020, mid-January 2021, and mid-December, 2021 respectively.

**Figure 4:**
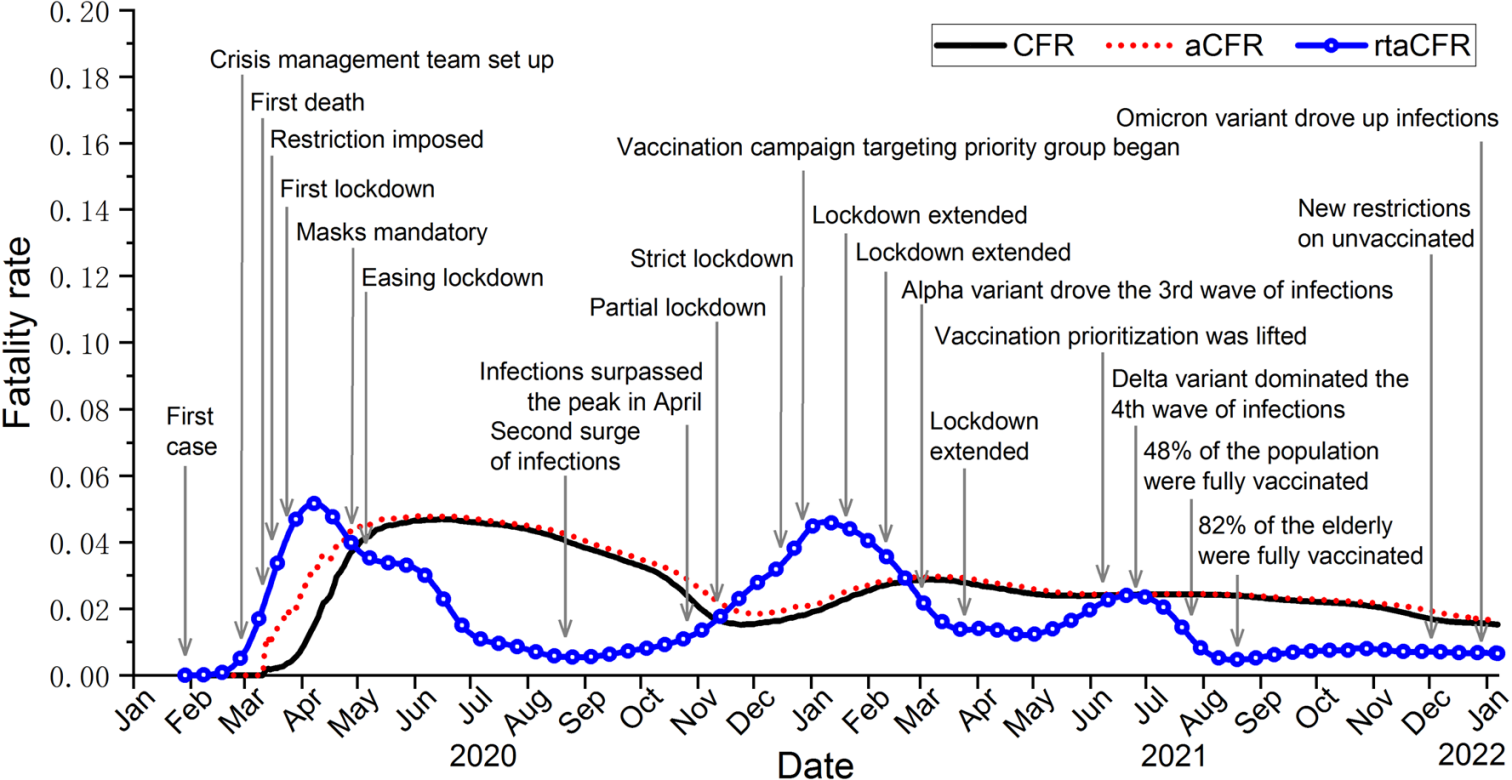
Estimates based on rtaCFR, aCFR and CFR for the outbreak of COVID-19 in Germany.

We apply the proposed method to assess the time-varying fatality rate of COVID-19 in Germany. We assume that *F* follows a gamma distribution with mean 15.43 days and shape parameter 2.03^20^. The piecewise constant black lines in Fig. 3a show the raw estimates of the fatality rate obtained using the fused lasso technique. The tuning parameter is set to be $\lambda =39000$, which gives the smallest residual sum of squares subject to $0 \le {\widehat{p}}(j)\le 1$ for all $j=1,\dots$. To gain an insight into the trend of fatality rate over time, the blue solid line shows the smoothed rtaCFR based on the original estimates using a Gaussian kernel density with a bandwidth equal to twenty days. To consider the robustness of the proposed method, we also fit the data using different Gamma distributions for *F* with a mean of 10 days and 18 days^9,21,22^, and the results are labelled as $\textrm{rtaCFR}_{-}$ and $\textrm{rtaCFR}_{+}$ in Fig. 3a, respectively. Analogous to what we observed in the simulation study, the results obtained are robust to these changes. Furthermore, to investigate the effect of the penalty term $\lambda$ in the fused lasso regression, Fig. 3b presents a sensitivity analysis showing the smoothed rtaCFR obtained using a range of different values for $\lambda$. We can see that as $\lambda$ increases, a larger penalty is imposed and the neighboring estimates are getting closer to each other across the time axis, yielding a smoother estimated curve for the real-time fatality rate. The sensitivity analysis demonstrates that the proposed method is quite robust against the changes in $\lambda$ as evidenced by Fig. 3b.

The estimates obtained based on three fatality rate estimators, together with the chronology of some important events and implemented measures, are displayed in Fig. 4 for comparison. Based on the rtaCFR, we can observe three peaks of fatality rates attained at around 0.052, 0.046 and 0.024 appearing in April 2020, January 2021 and July 2021, respectively, while the fatality rate maintains at low levels for the rest of the study period. The estimates obtained from CFR and aCFR differ significantly during the early stage of the epidemic, but are virtually identical from May 2020 onward. This is because, during the fast-growing epidemics, newly confirmed cases are almost negligible compared to cumulative cases with definitive outcomes, and thus the effect of the adjustment factor in the aCFR diminishes over time, leading to similar estimates from these two approaches. We can see that the CFR and aCFR estimates drive to a peak level close to 0.05 in May 2020, then they decline gradually until December 2020 and remain stable at around 0.03 throughout the whole period.

It is clear that the proposed fatality rate estimator, rtaCFR, is capable of providing timely information on the progress of the epidemic in Germany. The rtaCFR increased sharply with the surge of cases during the first wave, which may be due in part to inadequate preparation in the early stages of the outbreak, especially with the increased proportion of high-risk elderly among the cases reported at that time^29^. Fortunately, the reduction in cases resulting from lockdown and mitigation measures implemented since March 2020 kept hospitals from being overburdened, and the rtaCFR declined gradually soon after reaching its peak in April. Then the rtaCFR stayed at a low level until a second wave of infection hit Germany. We can see that the fatality rate kept increasing at the beginning of the second wave despite the implementation of the ‘lockdown light’, and it only started to decline when strict lockdown was reintroduced in January 2021. Indeed, it has been suggested that mild and long-term lockdown strategies had limited power and lowered efficiency in containing the disease in general as compared to strict and short-term lockdown^35^. Especially when infections overwhelm the healthcare system, the fatality rate can be surprisingly high due to inadequate medical resources^36,37^. The rtaCFR then declined gradually since January 2021 with a small peak of around 0.02 observed during the third wave in June 2021 dominated by the Delta variant. In the following months, the spread of the Delta and the novel Omicron variants drove the fourth wave of infections.

We can see that although the number of cases reached a record high during the fourth wave of infection, the estimated fatality rate was suppressed to a historically low level. The low fatality rate is probably due to the less lethal Omicron strain compared to the earlier COVID-19 variants^38^. In addition, as of mid- August 2021, 82% of the elderly that are at the highest risk of severe outcomes have been fully vaccinated^39^. The high vaccination coverage in the elderly population in late 2021 also helped to reduce hospitalization and death in the third and fourth waves, whereas the share of the older population among confirmed cases increased when the vaccines were not available during the first and second waves of infections in Germany^29^. These findings demonstrate the effectiveness of social restriction in the absence of vaccination, and the beneficial effects of vaccination that brings to the society in reducing the risks of infection and severe illness^40,41^.

## Discussion

A simple time-varying fatality rate estimator adjusted for reporting delays using only the aggregated count of cases and deaths is proposed in this paper. In the early stages of the COVID-19 outbreak, the daily case counts were the primary metrics by which health authorities determined which mitigation efforts were appropriate. However, because the new Omicron variant is less lethal but more contagious, with many infections having few or no symptoms, experts recommend shifting the focus from case counts to disease severity level, as it provides a more reliable picture of hospitalizations and deaths, especially in an era of vaccination^42,43^. After two years of struggling with the outbreak, many countries, including the United Kingdom, the United States, Singapore, South Korea, and much of Europe, are now considering a new phase of living with the virus. Rebooting the economies after a prolonged lockdown does not mean giving up disease control; instead, it places a greater demand on real-time monitoring of the epidemic so that decision on prompt implementation or relaxation of restrictions at the earliest possible point can be made to reduce hospitalizations and deaths while minimizing the impact of economic and social disruption^44^. Therefore, the fatality rate in real-time should instead be the leading indicator for the government when considering precautions.

The simulation study shows the strength of the proposed rtaCFR in terms of accuracy and sensitivity in capturing the changes in disease severity. Ignoring the delay from disease onset to death leads to an underestimation of the actual fatality in commonly used CFR estimator, and this effect is even more pronounced in fast-growing epidemics. More importantly, it could mislead the trends in fatality rate during ongoing epidemics. This was the case with the SARS outbreak in 2003, where an increasing trend in fatality rate is suggested by the CFR estimator but in fact, it was a false alarm caused by the way of calculating^6^. This shortcoming is evident in Fig. 1d–f of our simulation study, where CFR indicates an upward trend in disease severity, but the actual severity is decreasing. Furthermore, the CFR, calculated by the aggregated counts, is insensitive in capturing changes in fatality rate. This property is shared by aCFR estimator, where the detection of a change in fatality rate is inevitably delayed even if it could eventually match with the real trend. For emerging infectious diseases, falsified and delayed information may lead to a delayed response, an inappropriate policy decision, invalid reflection on the effectiveness of a certain implemented measure, and other disastrous consequences. We therefore propose to use the rtaCFR to capture the underlying trends in disease fatality during ongoing epidemics, whereas traditional CFR is more appropriate to serve as an indicator of overall disease severity after an epidemic ends.

The application to the COVID-19 data in Germany also demonstrates the usefulness of the proposed method in formulating public health policies during ongoing epidemics. We can see that the proposed rtaCFR is able to reflect changes in disease severity during a new wave of infections, whereas the other two commonly used estimators remain more or less constant in the late phase of the epidemic. Naturally, the upward trend in rtaCFR suggests that stricter public health measures should be implemented promptly, while the converse suggests that restrictions are sufficient or can be gradually eased. In addition to the mitigation measures and vaccine coverage, changes in the age structure of the reported cases over time are also thought to have driven trends in disease fatality. For example, studies found a noticeable increase in age among confirmed cases in spring and winter 2020 in Germany. The high fatality in the older age group may have contributed to an increase in population disease severity as indicated by rtaCFR during this period^22,29^. On the other hand, the increased mobility of young adults during the summer vacation led to a higher proportion of young patients recorded in autumn 2020 and 2021, which may partially explain the decline in rtaCFR after the peak of the first wave and the relatively low disease severity during the third and fourth waves^22,29,45^. Furthermore, gender differences in the severe outcome of COVID-19 were also demonstrated^46^.

Rising attention has been paid to monitoring the progression of the COVID-19 epidemic with the use of surveillance data in different regions, while considering the reporting delay in death figures. Zhao^47^ studied the estimation for instantaneous case fatality ratio in Canada using a maximum likelihood approach. The author assumed that the time interval between onset to death and the time interval between onset to confirmation are independent, and assumed a probability distribution to each of the time variables. In our work, we only model the time interval from onset to death and the assumption is modest in practice. Ko et al.^48^ studied the joint estimation of vaccine effectiveness against death and age-specific case fatality rate in Japan under the Bayesian paradigm. They assumed that the case fatality rate follows a beta distribution in the binomial process. In contrast to their approach, we do not impose a parametric assumption on the real-time fatality rate enjoying greater flexibility in modelling, but our method does not incorporate the explanatory variables.

It is worth noting that the infectious disease fatality rate is a composite measure, and in addition to the age and sex distribution of diagnosed cases we mentioned above, it can also be affected by many other factors, such as testing capacity, vaccination coverage, and even weather. A limitation of the study is that the effects of explanatory variables, such as age, sex and some healthcare burden indicators are not considered. Hence, it may not be fair to compare the trends of the fatality rates across multiple regions using the proposed estimator, especially when the population structures between regions being compared differ notably. Presumably, some standardization methods can be used to remove those effects by adjusting for differences in the age or gender distribution of the populations being compared if detailed demographic data are available. When numerous features are available in the study and the dynamics of the fatality over time is not the main focus, the lasso regression method, as a variable selection tool that allows sparsity in the covariate effects, has been widely applied to explore the most important factors affecting the level of COVID-19 fatality^49–51^. The fused lasso techniques on the time series fatality rate estimation in the literature are relatively limited but they have recently been applied to the susceptible-infected-recovered model tracking the transmission of the COVID-19 epidemic^52,53^. In this paper, variable selection is not the focus, and the fused lasso regression is simply used to incorporate temporal smoothness in the disease fatality estimates over time. We basically formulate the problem based on the frequentist approach as the fatality rate parameters are not random. It will be interesting to see if the proposed method can be extended to the Bayesian paradigm in the future.

Another limitation of the proposed method includes the potential ascertainment bias in the estimation of the fatality rate. Specifically, as only diagnosed cases are considered, bias will be introduced if the fatality rate of individuals identified with COVID-19 is not the same as that of undiagnosed individuals. The extent of this bias depends on the country-specific test coverage. In countries with widespread testing, such as China and Germany, this bias is negligible. Nevertheless, in countries where testing is less comprehensive, such as Italy and the United States, an upward bias may be incurred due to the under-detection of milder cases. In these circumstances, data about the testing volume and other additional information is required to estimate the ratio of under-reporting rate^54^. There is therefore an urgent call for a standardized data collection method by health authorities regarding the testing strategies, demographic characteristics and vaccination status of the cases to facilitate a better understanding of the trends in disease fatality. Despite the limitations, the proposed rtaCFR provides a valuable benchmark representing the risk of death under the influence of various factors during an ongoing epidemic. By monitoring the latest trend of fatality rate with the proposed method, the continuous decision-making process can be supported during the epidemic, even in a limited-data context. We hope that the proposed estimator can contribute to fighting against emerging epidemics worldwide.

## Data Availability

The datasets generated and/or analysed during the current study are available in the Johns Hopkins Coronavirus Resource Center repository, https://coronavirus.jhu.edu/region/germany.
